# Supplementary material for: Combined single-cell profiling of expression and DNA methylation reveals splicing regulation and heterogeneity
Source: Genome Biol. 2019 Feb 11;20:30. doi: 10.1186/s13059-019-1644-0 (PMC6371455; doi:10.1186/s13059-019-1644-0)
Supplement: Supplementary file 3 — Supplementary Results & Figure S1-S11. Supplementary results on the deep modeling of the splicing states in single cells and the Figures S1 to S11. (PDF 2190 kb) [file 13059_2019_1644_MOESM3_ESM.pdf]

Supplementary Results

Next to linear models we employed convolutional neural networks (CNN) to model the relationship between DNA methylation and splicing. Using this CNN approach we aimed to get more insights into the complex relationship between DNA methylation and the increased or decreased splicing rates.

For each exon, a sequence window of ±400 bp around the centre of the alternative exon was used to train a CNN that predicts inclusion (PSI ≥ 1/3) and exclusion (PSI < 1/3) of the alternative exon. We chose this sequence region since the linear models revealed the relevance of the alternative exon when predicting splicing rates. In addition, the alternative exon has been used in previous analyses [1, 2]]. As most exons are shorter than 200 bp [3], the chosen sequence region mostly covers the whole exon as well as the most relevant regions from the adjunctive introns. Besides using a four-letter code for the four DNA bases, we also integrated methylation data in a five-letter code that includes methylated ('M') and un-methylated ('U') cytosines (Methods). The sequences of both four-letter and five-letter code were one-hot encoded and multiplied with the conservation score of the region, as derived from BRIE.

We obtained scores for each position of the sequence by a sliding window approach of 58 filters of length 10 bp. A bias of 1 was added. The rectified linear unit (relu) was used as activation function. Subsequent layers include a maximum pooling step with pooling length of 8 bp and a hidden layer with 50 nodes.

The cassette exons were split into training, validation and test set (60%, 20%, 20%). The model was trained until weight convergence (patience: 3 iterations, maximum: 10 iterations).

The five-letter code model performed slightly better than the four-letter code model (AUC=0.912 and 0.908, respectively) (**Figure S6**). Similar to the linear models that predict inclusion and exclusion of the alternative exon, the methylation information does not improve predictability of splicing much. The CNN performances are comparable to state-of-the-art models that were applied to bulk data [1, 2]. To our knowledge this is the first CNN trained on single-cell splicing data, and that allows inclusion of DNA methylation information to improve prediction of splicing levels.

Here we show that a deep neural network can be used to predict single-cell splicing with good performance. The inclusion of methylation information by designing a five-letter alphabet that includes methylated and unmethylated cytosines led to a minimal improved prediction of splicing levels. To make this model, accessible to the scientific community we uploaded it to the kipoi.org model zoo[4], so it can be employed to make regulatory gene predictions using genomic and epigenomic variation.

References

1. Wainberg M, Alipanahi B, Frey B. Does conservation account for splicing patterns? BMC Genomics. 2016;17:787. doi:10.1186/s12864-016-3121-4.
2. Xiong HY, Alipanahi B, Lee LJ, Bretschneider H, Merico D, Yuen RKC, et al. RNA splicing. The human splicing code reveals new insights into the genetic determinants of disease. Science. 2015;347:1254806. doi:10.1126/science.1254806.
3. Sakharkar MK, Chow VTK, Kangueane P. Distributions of exons and introns in the human genome. In Silico Biol. 2004;4:387–93. doi:2004040032 [pii].
4. Kreuzhuber R, Israeli J, Xu N, Cheng J, Kundaje A. Kipoi : accelerating the community exchange and reuse of predictive models for genomics. 2018;:1–31.

Supplementary figures

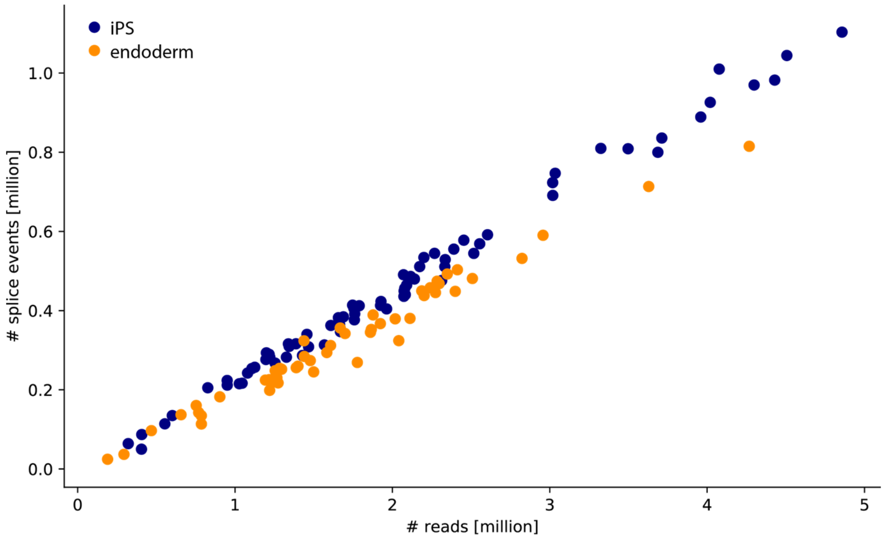

Fig S1. Number of detected exon skipping events (minimum coverage five reads) versus the number of reads per single cell, for iPS (blue) and endoderm (orange) cells, respectively.

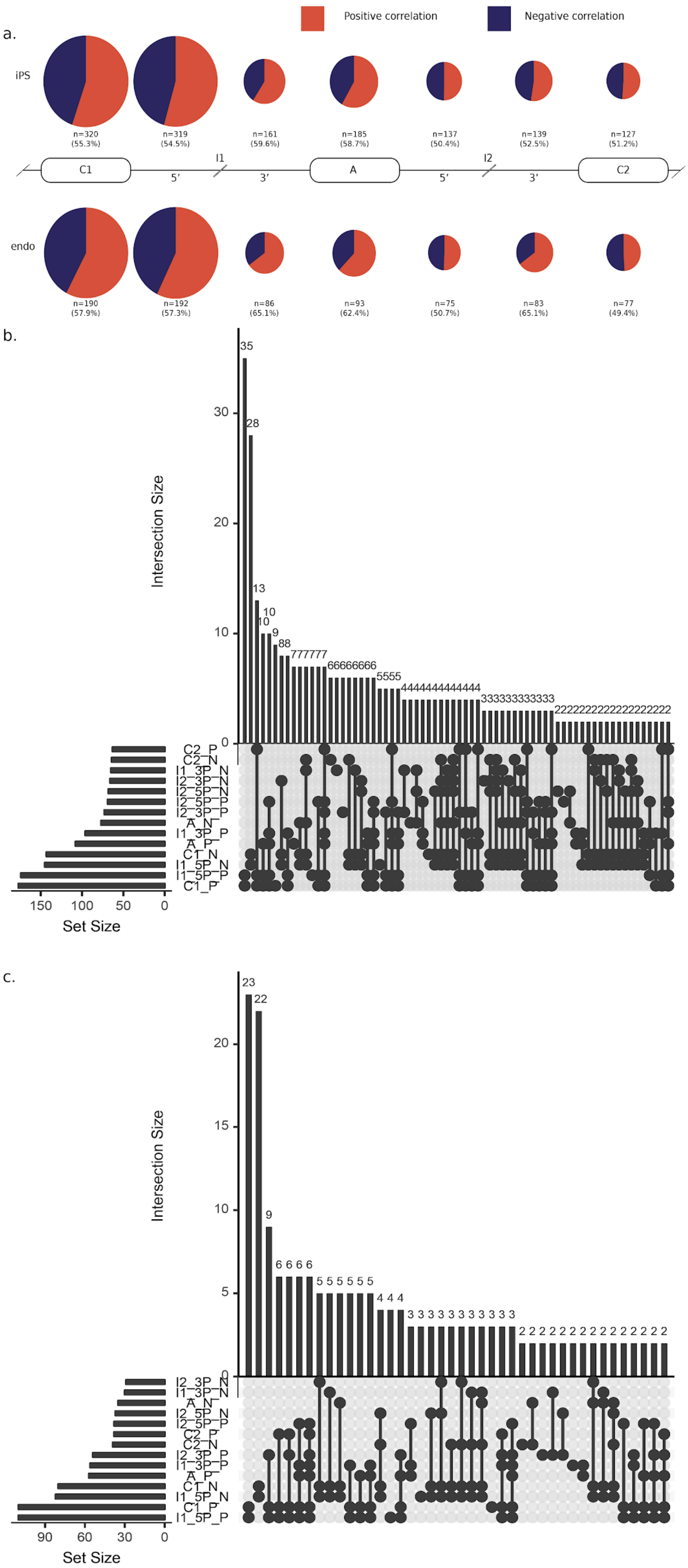
a.
Positive correlation
Negative correlation
iPS
n=320 (55.3%)
n=319 (54.5%)
n=161 (59.6%)
n=185 (58.7%)
n=137 (50.4%)
n=139 (52.5%)
n=127 (51.2%)
C1
5’
I1
3’
A
5’
I2
3’
C2
endo
n=190 (57.9%)
n=192 (57.3%)
n=86 (65.1%)
n=93 (62.4%)
n=75 (50.7%)
n=83 (65.1%)
n=77 (49.4%)
b.
Intersection Size
C2_P
C2_N
I1_3P_N
I2_3P_N
I2_5P_N
I2_5P_P
I2_3P_P
A_N
I1_3P_P
A_P
C1_N
I1_5P_N
I1_5P_P
C1_P
Set Size
c.
Intersection Size
I2_3P_N
I1_3P_N
A_N
I2_5P_N
I2_5P_P
C2_P
C2_N
I2_3P_P
I1_3P_P
A_P
C1_N
I1_5P_N
C1_P
I1_5P_P
Set Size

Fig S2. Additional results for the single-exon association analysis between DNA methylation and splicing. **a.** Distributions of positive and negative associations between DNA methylation and splicing per sequence context for iPS and endoderm cassette exons, respectively. **b.** Upset plot showing the overlap between associations found in multiple sequence contexts including effect directions (in iPS cells). Unique associations for a specific set are not shown. **c.** Upset plot showing the overlap between associations found in multiple sequence contexts including effect directions (in endoderm cells). Unique associations for a specific set are not shown.

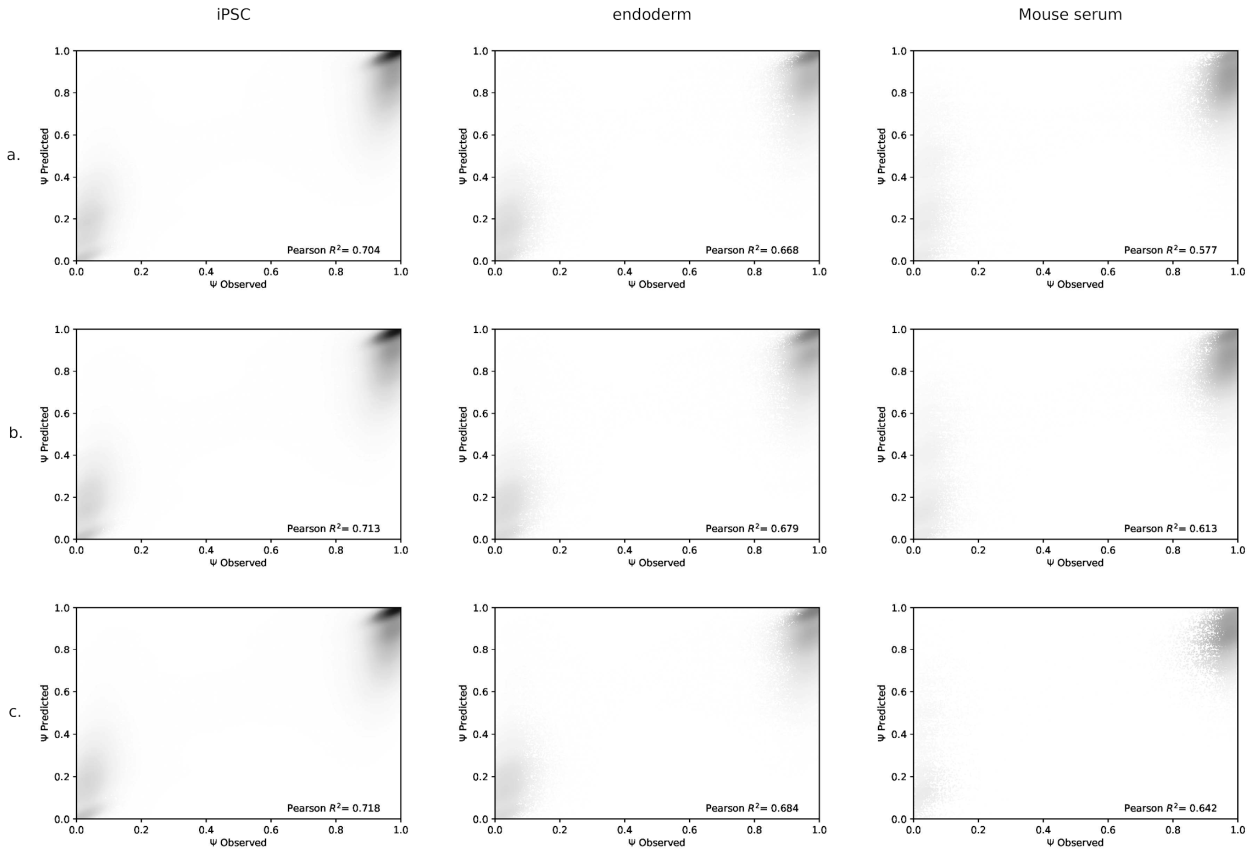

Fig. S3. Scatter plots between observed and predicted single-cell splicing rates and Pearson r^2 of splicing predictions for iPS, endoderm and mouse cells (columns). The three alternative regression models are based on different predictive features (rows): **a.** Only genomic features. **b.** Genomic features and mean methylation information across cells. **c.** Genomic features and cell-matched methylation information.

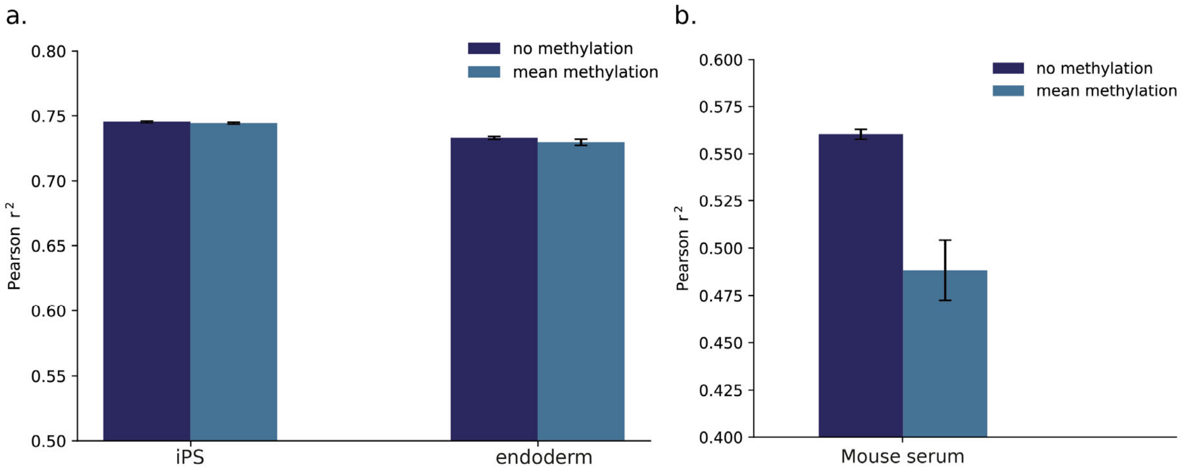

Fig.S4. Pearson r^2 of the linear models predicting pseudo-bulk splicing by aggregating read counts across cells. **a.** Performance for iPS (left) and endoderm cells (right). The model that includes DNA methylation information (r^2=0.744, r^2=0.729 at iPSC and endoderm) yields comparable prediction accuracy to the model without DNA methylation information (r^2=0.745, r^2=0.733, respectively). **b.** Performance for mouse cells. The model that includes DNA methylation information (r^2=0.56) performs worse in terms of prediction accuracy than model without DNA methylation information (r^2=0.489).

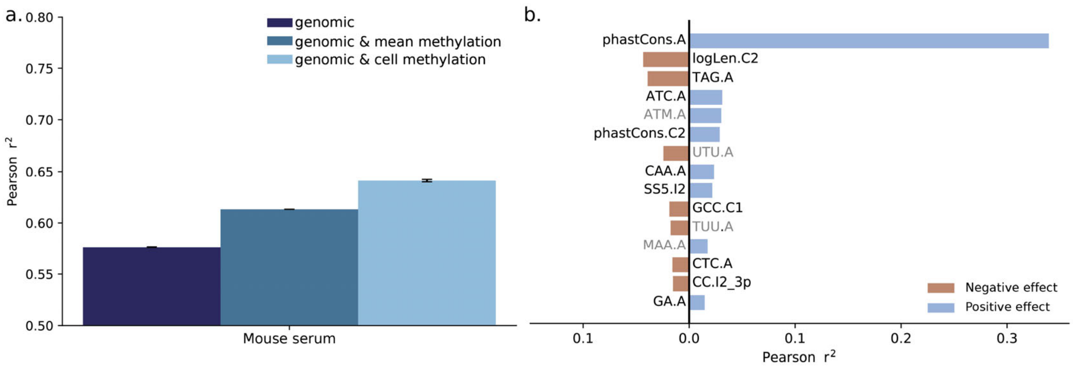

Fig.S5. Regression-based prediction of single-cell splicing variation in mouse cells. **a.** Prediction accuracy (r^2 based on 10-fold CV) of alternative regression models for predicting single-cell splicing rates. The genomic model is based on sequence k-mers, conservation scores and lengths of contexts (size of the cassette exon, length of flanking introns) as features (genomic features, dark blue). Other models account for average methylation rates across cells (genomic & mean methylation, blue), or cell-specific methylation rates (genomic & cell methylation, light blue). Error bars denote plus or minus one standard deviation across four repeat experiments. **b.** Correlation between features predictive of splicing and PSI. The features are ranked by relevance for predicting splicing as determined by single-feature regression models trained on single cells. Positive correlation shows increased alternative exon inclusion with an increased feature score.

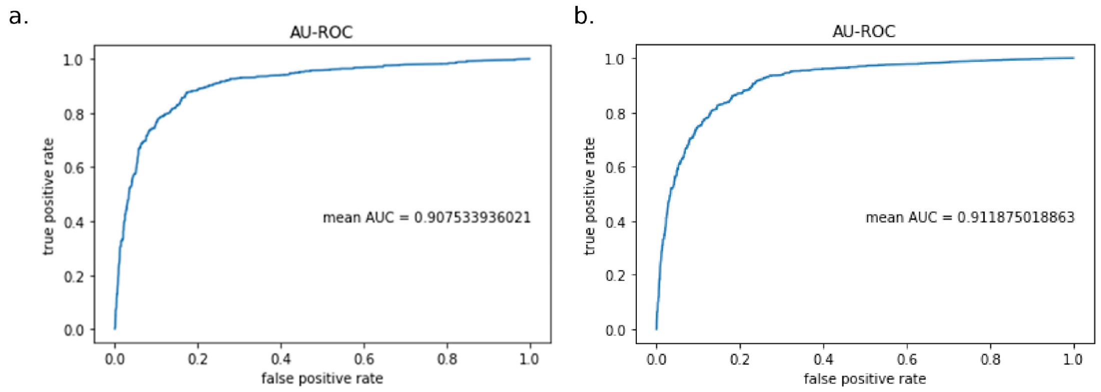

Fig. S6. AU-ROC curves and AUCs of a CNN when predicting splicing from genomic sequences in iPS cells. **a.** Performance of the CNN based on only genomic information (four-letter code model). **b.** Performance of the CNN based on genomic and methylation information (five-letter code model).

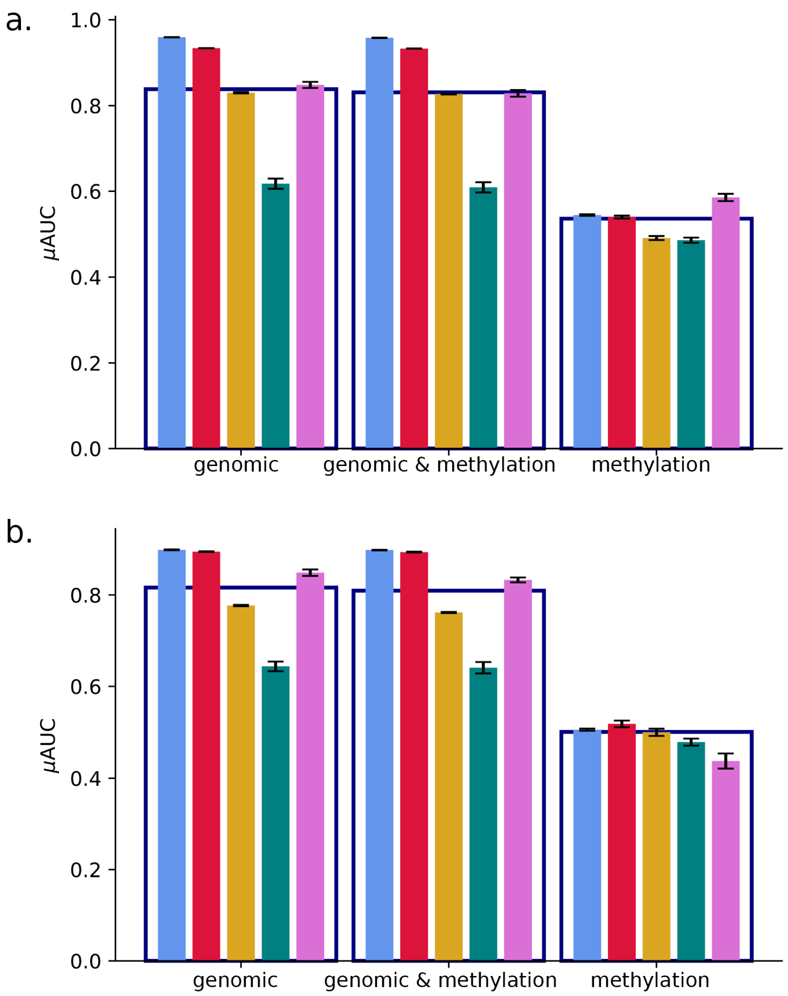

Fig. S7. Prediction performance of alternative regression models for each splicing category in **a.** endoderm and **b.** mouse cells. The model either considers genomic features ('genomic'), genomic and all DNA methylation features ('genomic & methylation') or only DNA methylation features ('methylation'). The genomic model includes k-mers, conservation scores and region lengths, the genomic and methylation model additionally includes DNA methylation features. The methylation model includes average DNA methylation features per sequence context. Splicing categories are coded in color as in **Fig. 3a**. Error bars denote plus or minus one standard deviation across four repeat experiments.

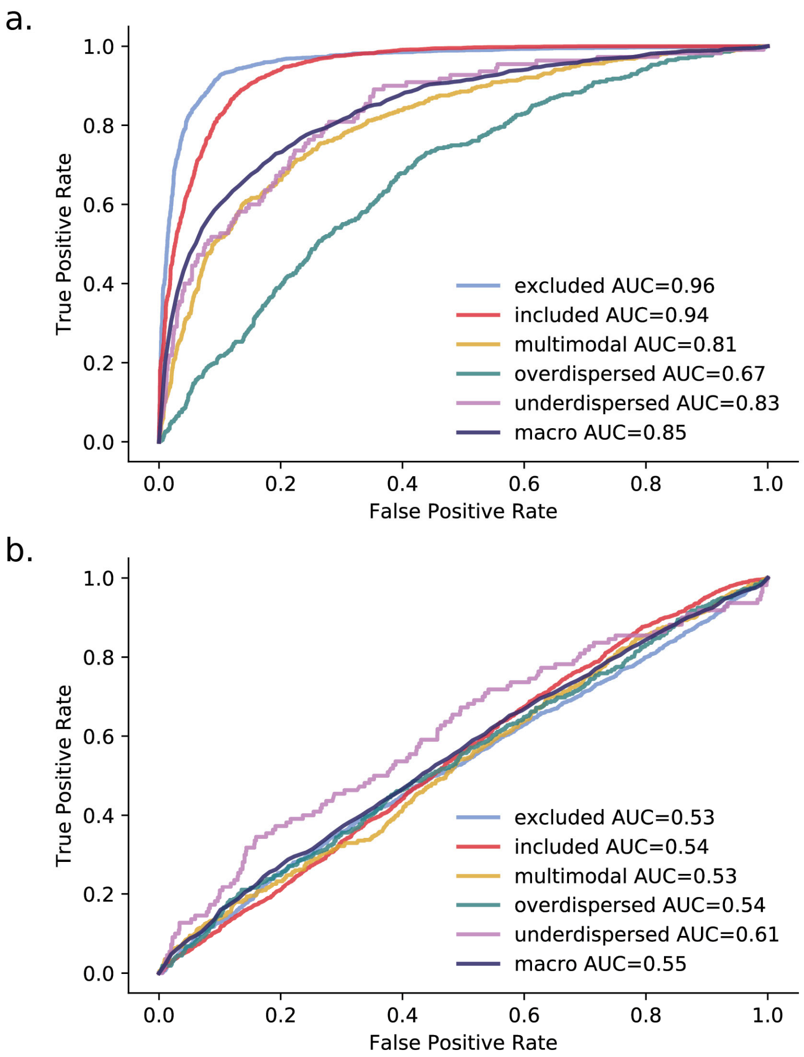

Fig. S8. AU-ROC curves and AUCs when predicting splicing categories with one-vs.-rest logistic ridge regression models and different sets of predictive features within iPS cells. The performances of each individual splicing category and the macro performance across all categories are shown. **a.** Genomic and DNA methylation features. **b.** Mean methylation features.

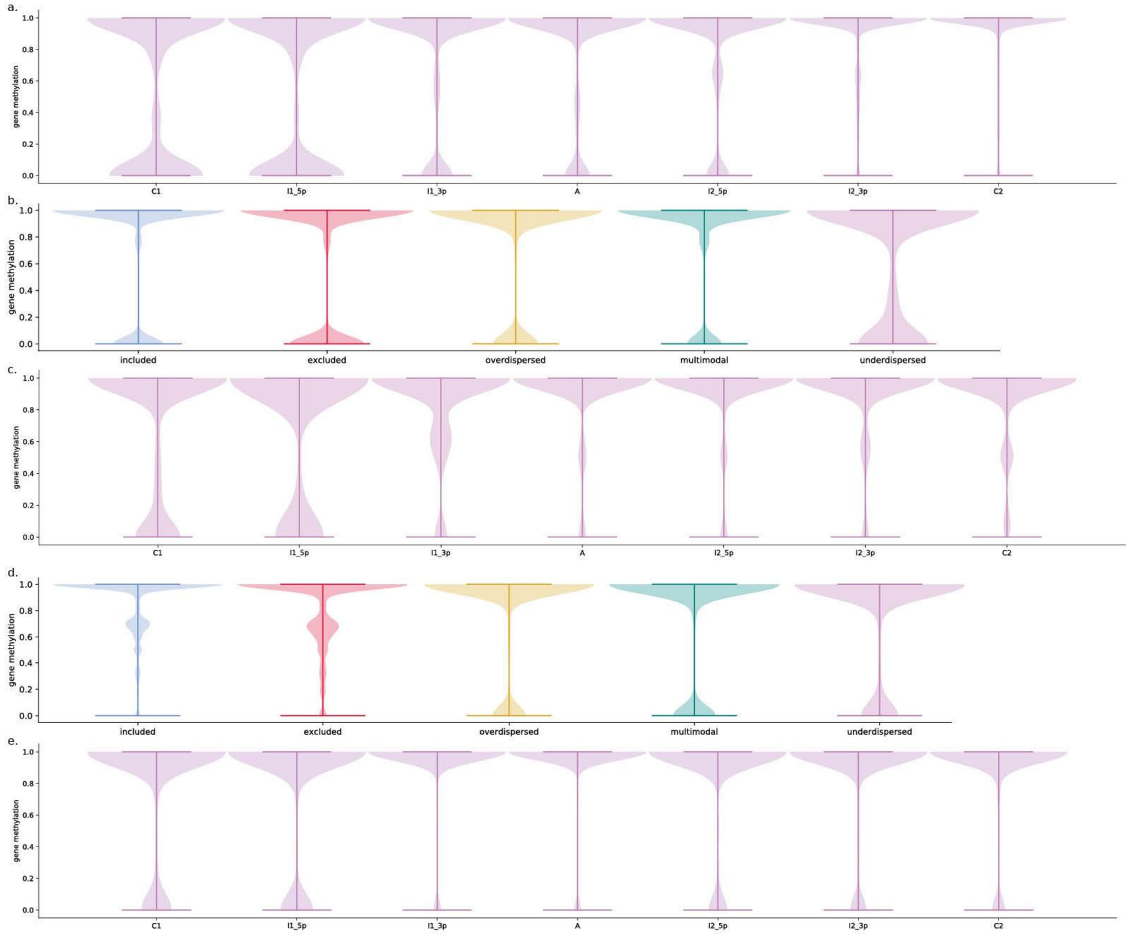

Fig. S9. The distribution of DNA methylation rates per sequence context or categories. **a.** The methylation rates in iPS for the overdispersed cassette exons across context, **b.** The methylation rates of the C1 region in endoderm across the splicing categories, **c.** The methylation rates in endoderm for the overdispersed cassette exons across context, **d.** The methylation rates of the C1 region in mouse cells across the splicing categories and **e.** The methylation rates in mouse cells for the overdispersed cassette exons across context.

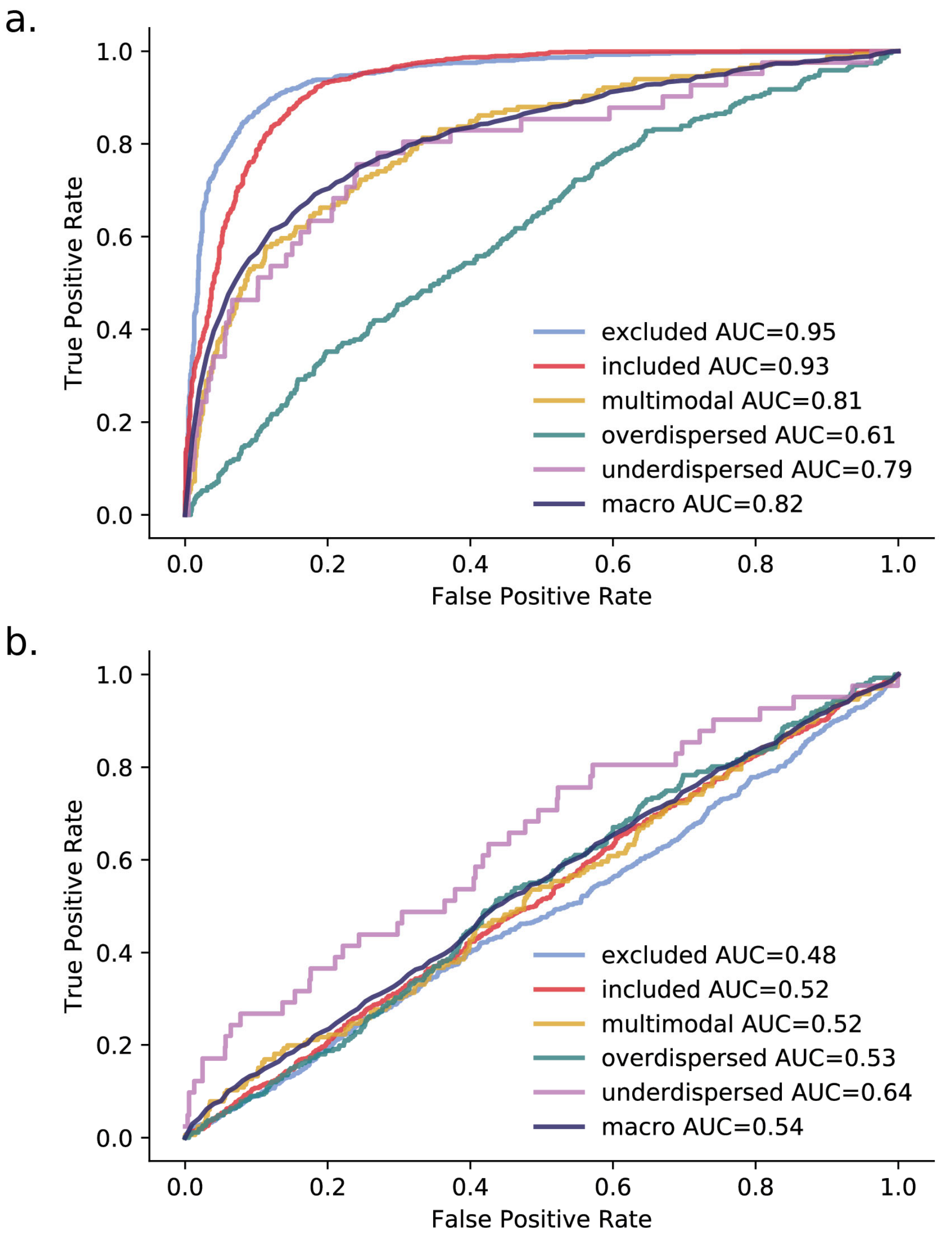

Fig.S10. AU-ROC curves and AUCs when cross-predicting splicing categories across tissue types. Performance of predicting categories of exclusive iPS exons with a one-vs.-rest logistic ridge regression trained on all endoderm exons is shown. The performances of each individual splicing category and the macro performance across all categories are shown. Different sets of predictive features are used, namely **a.** genomic features, and **b.** genomic and methylation features.

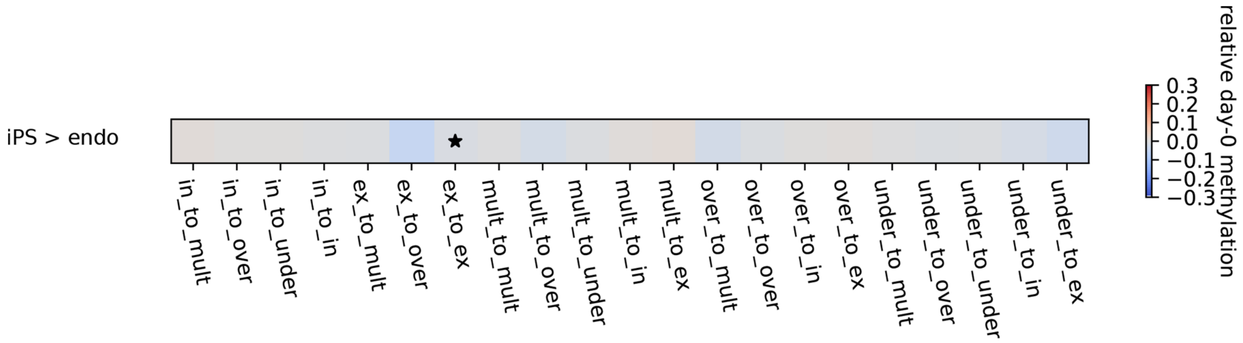

Fig. S11. iPS cell versus endoderm cell DNA methylation rate differences of the splicing category-switching cassette exons. A significant decrease in DNA methylation rate is observed between the excluded iPS and excluded endoderm categories. [included (in), excluded (ex), multimodal (mult), overdispersed (over), underdispersed (under)]
